# Phylogenetic analysis and accessory genome diversity reveal insight into the evolutionary history of *Streptococcus dysgalactiae*

**DOI:** 10.3389/fmicb.2022.952110

**Published:** 2022-07-19

**Authors:** Cinthia Alves-Barroco, Patrícia H. Brito, Ilda Santos-Sanches, Alexandra R. Fernandes

**Affiliations:** ^1^Applied Molecular Biosciences Unit (UCIBIO), Departamento de Ciências da Vida, NOVA School of Science and Technology, Costa da Caparica, Portugal; ^2^i4HB, Associate Laboratory – Institute for Health and Bioeconomy, Faculdade de Ciências e Tecnologia, Universidade NOVA de Lisboa, Costa da Caparica, Portugal; ^3^NOVA Medical School, Faculdade de Ciências Médicas, Universidade NOVA de Lisboa, Lisbon, Portugal

**Keywords:** SDSD and SDSE, core-genome, accessory genome, *Streptococcus dysgalactiae*, prophages regions

## Abstract

Streptococcus dysgalactiae (SD) is capable of infecting both humans and animals and causing a wide range of invasive and non-invasive infections. With two subspecies, the taxonomic status of subspecies of SD remains controversial. Subspecies *equisimilis* (SDSE) is an important human pathogen, while subspecies *dysgalactiae* (SDSD) has been considered a strictly animal pathogen; however, occasional human infections by this subspecies have been reported in the last few years. Moreover, the differences between the adaptation of SDSD within humans and other animals are still unknown. In this work, we provide a phylogenomic analysis based on the single-copy core genome of 106 isolates from both the subspecies and different infected hosts (animal and human hosts). The accessory genome of this species was also analyzed for screening of genes that could be specifically involved with adaptation to different hosts. Additionally, we searched putatively adaptive traits among prophage regions to infer the importance of transduction in the adaptation of SD to different hosts. Core genome phylogenetic relationships segregate all human SDSE in a single cluster separated from animal SD isolates. The subgroup of bovine SDSD evolved from this later clade and harbors a specialized accessory genome characterized by the presence of specific virulence determinants (e.g., *cspZ*) and carbohydrate metabolic functions (e.g., fructose operon). Together, our results indicate a host-specific SD and the existence of an SDSD group that causes human–animal cluster infections may be due to opportunistic infections, and that the exact incidence of SDSD human infections may be underestimated due to failures in identification based on the hemolytic patterns. However, more detailed research into the isolation of human SD is needed to assess whether it is a carrier phenomenon or whether the species can be permanently integrated into the human microbiome, making it ready to cause opportunistic infections.

## Introduction

The taxonomic status of subspecies *Streptococcus dysgalactiae* (SDSD) remains controversial despite years of debate and reclassifications. Based on whole-cell-derived polypeptide patterns, chemotaxonomic and phenotypic examination, Vandamme et al. (1996) proposed the criteria for the classification of *Streptococcus dysgalactiae* subspecies, in which subspecies *dysgalactiae* includes isolates from the host animal, while the subspecies *equisimilis* (SDSE) includes isolates from the human host. Posteriorly, Vieira et al. (1998) classified the two subspecies SDSD and SDSE, based on hemolytic patterns including α-hemolytic and β-hemolytic isolates, respectively (Vieira et al., 1998).

The subspecies SDSE, initially regarded as human commensal bacteria (Suzuki et al., 2011), is now recognized to be an important human pathogen, causing several infections, including bacteremia, cellulitis, endocarditis, peritonitis, septicemia, pneumonia, pharyngitis, and toxic shock syndrome, similar to those caused by *S. pyogenes* (Brandt and Spellerberg, 2009; Bruun et al., 2013; Hagiya et al., 2013; Fuursted et al., 2016). Additionally, β-hemolytic isolates from animal hosts have been frequently identified as SDSE (Acke et al., 2015; Pinho et al., 2016; Oh et al., 2018, 2020; Porcellato et al., 2021).

The subspecies SDSD has been considered strictly an animal pathogen, and it is commonly associated with bovine mastitis (Rato et al., 2013) and infectious arthritis in sheep (Smistad et al., 2021) and septicemia in vampire bats (Mioni et al., 2018). SDSD has been considered an important pathogen in aquaculture systems worldwide (Maekawa et al., 2020; Hawke et al., 2021). Phylogenetic studies based on the *sodA* gene revealed that isolates from human blood cultures are closely related to SDSD from the fish host, suggesting that SDSD represents an important potential causative agent of zoonoses (Koh et al., 2009, 2020).

Recently, we have reported that bovine SDSD can adhere to and internalize human cells, including human epidermal keratinocyte cells (Alves-Barroco et al., 2018), suggesting that bovine SDSD may cause skin/soft tissue infections, and this might be an important mechanism in the pathogenesis of human cellulitis. In fact, human cellulitis by SDSD, although still rare, has been reported (Koh et al., 2009; Park et al., 2012; Jordal et al., 2015; Chennapragada et al., 2018; Nathan et al., 2021).

Differences in the adaptation of SDSD to humans and bovines are still unknown. It is critical to understand further the pathogenic potential of the most clinically significant strains and their epidemiology. Previous studies have reported the presence of phage-carried virulence genes of *S. pyogenes* among SDSD strains from bovine mastitis, such as the streptococcal pyrogenic exotoxin genes and DNases extracellular (Rato et al., 2011; Abdelsalam et al., 2013; Alves-Barroco et al., 2021). It was suggested that the presence of these virulence genes of *S. pyogenes* in SDSD contributes to the increased virulence potential of this subspecies (Rato et al., 2011).

In routine laboratory practice, SDSD and SDSE have been distinguished based on their hemolytic properties on blood agar. However, failures in identification were shown based on hemolytic patterns since SDSD can also produce β-hemolysis (Jensen and Kilian, 2012).

The purpose of this study was to analyze whole-genome sequences of SD from the National Center for Biotechnology Information that included the previously described SDSD and SDSE and four new bovine SDSD strains to understand the molecular characteristics associated with the ability to infect different hosts. More specifically, our purpose is to reexamine the phylogenetic relationship among SD strains from different hosts (human and non-human) using the single-copy core genome and present comparative analyses of accessory genes that influence the adaptation to different hosts.

## Materials and methods

To understand the complete genomic repertoire, we analyzed 102 different SD genomes from National Center for Biotechnology Information (all genome assemblies presented were downloaded on January 2021) (Supplementary Table S1). Four alpha-hemolytic SDSD bovine strains previously studied (Rato et al., 2010; Alves-Barroco et al., 2021) from Portugal were included in the study. The classification at the subspecies level and the source, presented in Supplementary Table S1, follow information from databases and previous publications.

### Genomic DNA extraction, sequencing, assembly, and annotation

Genomic DNA was extracted according to Alves-Barroco et al. (2021). Each gDNA was quantified in a Nanodrop Spectrophotometer (ThermoFisher Scientific, Waltham, MA, United States) (VSD9 – 29.6 ng/μL; VSD16 – 41.2 ng/μL; VSD22 – 33.0 ng/μL; VSD43 – 34.8 ng/μL). The integrity samples were confirmed by gel electrophoresis [1% (w/v) agarose], and images were captured using the Gel Doc XR system and Quantity One 1-D analysis software (Bio-Rad, United States). The samples fulfilled all the conditions and therefore proceeded for library construction and sequencing. The generated DNA fragments (DNA libraries) were sequenced with the Illumina Novaseq platform using 150-bp paired-end sequencing reads at STAB VIDA, Caparica, Portugal. The quality of the produced data was determined by Phred quality score at each cycle (position in read). The plot containing the average quality at each cycle was created with the FastQC tool (Andrews, 2010).

Sequencing reads were quality-trimmed using the CLC Genomic Workbench 20.1 (Qiagen, Denmark^1^) with default parameters for the removal of low-quality sequences. Preprocessed reads were *de novo* assembled with the CLC Genomic Workbench, using the default settings and a minimum contig length setting of 500 bp. The complete genome sequence data have been submitted to the NCBI and have been deposited at DDBJ/ENA/GenBank under accessions: JAJSPH000000000 (SDSDVSD9), JAJSPI000000000 (SDSDVSD16), JAJSPJ000000000 (SDSDVSD22), and JAJSPK000000000 (SDSDVSD45).

Genome sequences were annotated using the Rapid Annotation using Subsystem Technology (RAST) server (Aziz et al., 2008). The RASTtk pipeline applies Prodigal and Glimmer3 prokaryote gene prediction tools, annotates protein-encoding genes’ hypothetical proteins with k-mers, and performs a basic gene overlap removal. RAST bases its genome identifiers on NCBI taxonomy IDs. The RAST annotation scheme was performed using the RASTtk pipeline. Additionally, we selected the option to automatically resolve automatic annotation (such as gene candidates overlapping RNAs or genes embedded inside other genes).

### Pangenome analysis of *Streptococcus dysgalactiae* genomes

The Spine software (v0.2.3) (Ozer et al., 2014) was used to identify *S. dysgalactiae’s* conserved core genome sequences (genomic sequences present in 100% of the strains) using SDSE NCTC6403 as the reference genome sequence. Alignments with at least 85% sequence identity among *S. dysgalactiae* strains were considered homologous. For a comparative analysis, the core genome using four strains of *S. pyogenes* used as outgroups was also estimated (Supplementary Table S1). Only alignments with at least 70% sequence identity were considered homologous.

#### Core genome phylogenetic analysis

Nucleotide sequences of single-copy protein-encoding genes were identified and selected, creating alignment blocks using CLC Genomic Workbench. Subsequently, the phylogenetic tree was inferred using maximum-likelihood in IQ-TREE v2.0.3 (Nguyen et al., 2015). Modelfinder (Kalyaanamoorthy et al., 2017) was used to determine the best model, and branch support was estimated using fast bootstrap approximation with NNI optimization and 1,000 replicates (Hoang et al., 2018). For each pair of core *S. dysgalactiae* genomes in the alignment, the average nucleotide identity (ANI) was performed using CLC Genomic Workbench. Clustering of the pairwise comparison of ANI results was constructed using Euclidian distances.

#### Accessory genome prediction and characterization

The AGEnt was used for identifying accessory genomic elements (AGEs) in only the genomes *of S. dysgalactiae* using an *in silico* subtractive hybridization approach against a core genome generated using the Spine algorithm and the default threshold for alignments of 85% sequence identity over at least 100 bp. Annotation of the core and accessory genome was performed using the Rapid Annotations using Subsystems Technology (RAST) (Aziz et al., 2008).

The CCMetagen analysis tool was used to determine the probable origin of the accessory genome of *S. dysgalactiae* strains (Marcelino et al., 2020). Alignment of accessory genes against the GenBank database using BLAST was performed to trace homolog genes. For comparative analysis of the accessory genome of *S. dysgalactiae* infecting different hosts, genes present in only single or less than 5% genomes (for each group of isolates from different hosts) were not considered due to their higher probability of being false positives.

CLC Genomics Workbench was used to analyze predicted proteins and find suitable structures for M-like proteins. BLAST against M-like protein structure sequence database was carried out by the Find and Model Structure. BLAST hits with an identity to the query sequence lower than 40% were removed since they most likely would result in inaccurate models. Protein Data Bank (PDB) structures with a resolution lower than 4 Å were removed since they cannot be expected to represent a trustworthy atomistic model. A template quality score is calculated for the available structures found for the query sequence. The score’s purpose is to consider both their quality and homology to the query sequence.

#### Prophage identification

PHASTER software (Arndt et al., 2016) was used to screen for prophage-specifying DNA regions within the genomes of available *Streptococcus dysgalactiae*. Based on the completeness and potential viability of identified prophages, these were identified as “intact,” “questionable,” or “incomplete” prophages. Intact prophages were manually analyzed to confirm the presence of all genes required to produce a functional phage particle, including genes encoding the following: left attachment site (attL); lysogeny; DNA replication; transcriptional regulation; head; tail; lysis modules; and right attachment site (attR) (Canchaya et al., 2003). The intact prophage sequences were extracted and used as the query in BLAST searches against the NCBInr database to screen for the presence of similar prophages in all available genomes. Since several of the SD genomes analyzed are contigs, some regions of prophages may have split into different contigs, and only complete sequences of intact prophages were analyzed. For this analysis, only overlap and identity greater than 60 and 80%, respectively, were considered.

## Results

### Characterization of four new genomes of bovine SDSD

This study provides new complete genome sequences for four SDSD isolates, Vet *Streptococcus dysgalactiae* (VSD) was isolated from bovine mastitis in a Portuguese dairy herd (Supplementary Table S1). For comparative purposes, we provide the same information for representative SDSD genomes available at NCBI that are associated with infections of different hosts (human, fish, and bovine). The overview of the general characteristics of the new SDSD genomes is shown in Table 1 and Supplementary Table S2. The whole-genome size of the VSD isolates had an average of 2.05 MB with an average G + C content of 39.6% (Supplementary Table S2). On average there are 2,123 coding sequences (CDSs), representing 95.6, 91, 93.5, and 93.6% of the entire genome of VSD9, VSD16, VSD23, and VSD45, respectively. Of the protein-coding genes, 83% of the CDSs were successfully assigned to a functional category of the cluster of orthologous groups (COG), and approximately 16% of the chromosomal products are hypothetical proteins. The results obtained for the new genomes are similar across all the SDSD genomes that were analyzed (Table 1 and Supplementary Table S2). However, some interesting genome features tend to differentiate the strains depending on the infected host, namely, strains that infect the bovine host tend to have more genes associated with carbohydrate, fatty acids, lipids, and isoprenoids metabolism, and respiration than strains that infect humans and fish, but a lower number of genes associated with virulence and defense systems, iron acquisition, metabolism, and membrane transport (Supplementary Figure S1).

**TABLE 1:** Overview of the general characteristics SDSD genomes.

Subsystem feature counts	Bovine (10)	Fish (2)	Human (3)
**Amino Acids and Derivatives**	100 ± 3.2	98 ± 1	98 ± 3
Carbohydrates *	158 ± 6.7	132 ± 3.5	133 ± 4.0
**Cell Division and Cell Cycle**	4 ± 0.0	4 ± 0.0	4 ± 0.0
**Cell Wall and Capsule**	41 ± 1.6	42 ± 1	39.3 ± 1
**Cofactors. Vitamins. Prosthetic Groups. Pigments**	71 ± 0.8	71 ± 1	72 ± 3
**DNA Metabolism**	55 ± 4.0	57 ± 3	55 ± 6
Fatty Acids. Lipids. and Isoprenoids*	57 ± 0.0	41 ± 4	38.3 ± 3
Iron acquisition and metabolism*	20 ± 3.6	25 ± 1	27.3 ± 4
Membrane Transport*	22 ± 0.6	26 ± 2	26 ± 1
**Metabolism of Aromatic Compounds**	2 ± 0.0	2,5 ± 1	2.3 ± 0.0
**Miscellaneous**	10 ± 0.0	10,5 ± 1	11 ± 1
**Nucleosides and Nucleotides**	81 ± 1.9	79 ± 3	82.6 ± 5
**Prophages**	21 ± 7.3	30 ± 11	14.3 ± 6
**Protein Metabolism**	107 ± 6.1	112,5 ± 1	109 ± 6
**Regulation and Cell signaling**	17 ± 0.6	17 ± 1	17.3 ± 0.0
Respiration*	26 ± 3.5	20 ± 1	19.6 ± 1
**RNA Metabolism**	32 ± 2.0	30,5 ± 1	30.6 ± 0.0
**Stress Response**	16 ± 0.4	15,5 ± 1	16 ± 0.0
Virulence. Disease and Defense *	33 ± 3.7	40.5 ± 5.5	42.3 ± 3

*Mean and standard deviation of the values observed in the different SDSD genomes listed in Supplementary Table S1.

Bovine isolates: VSD9, VSD16, VSD22, VSD45, NCTC4669, NCTC4670, NCTC4671, NCTC13731, FDAARGOS1157, and ATCC27957. Fish isolates: Kdys0611 and STREP9715. Human isolates: DB4999805, DB6070515, and DB5399317.

### Pangenome diversity and phylogenetic analysis of *Streptococcus dysgalactiae*

The *S. dysgalactiae* pangenome was inferred from a dataset of 106 whole-genome sequences. In total, it comprises 6,745,861 bp, which includes 6,632 and 1,134 (1,542,420 bp) protein-coding sequences identified as accessory and core genomes, respectively.

**FIGURE 1:**
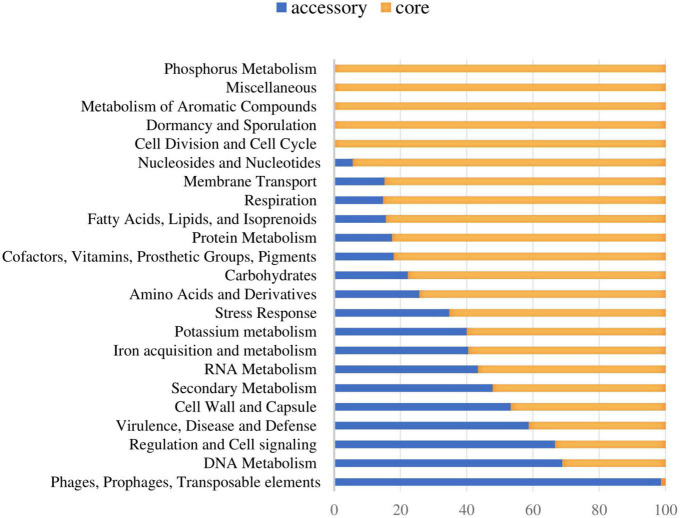
Distribution of the core and accessory genes in different functional categories investigated in *S. dysgalactiae* genomes.

The functional distribution of system categories between the accessory and core genomes of *S. dysgalactiae* is shown in Figure 1. The highest percentage of genes associated with the accessory genome was attributed to the category of prophages and transposable elements, followed by DNA metabolism, virulence and defense, regulation, and cell signaling. As expected, genes belonging to division and cell cycle categories and dormancy and sporulation were entirely attributed to the core genome. Identification of protein-coding sequences of the core genome is presented in Supplementary Table S3.

**FIGURE 2:**
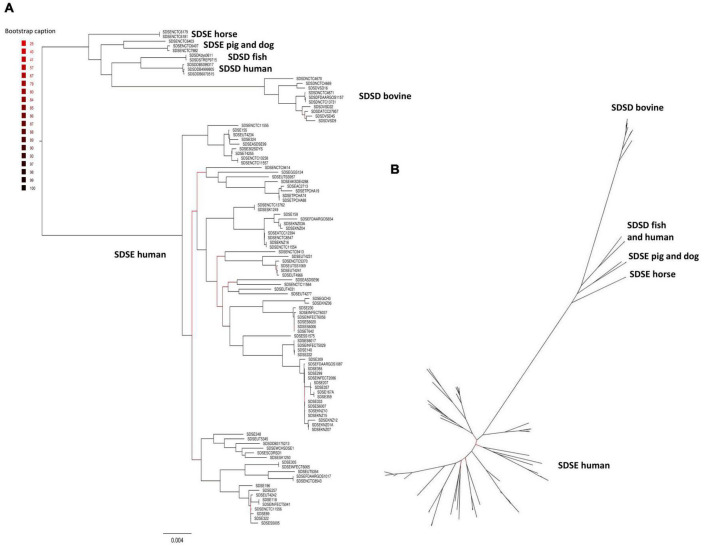
Phylogenetic analysis of the *S. dysgalactiae* single-copy core genome. **(A)** The tree was rooted on the largest branch as the closest outgroup species. **(B)** Phylogenetic unrooted tree relationships. The evolutionary history was inferred using the maximum likelihood analysis of the alignment of 1,134 single-copy core protein-coding sequences shared by 106 *S. dysgalactiae* strains. Bootstrap support values were calculated from 1,000 replicates. A phylogenetic tree was generated using IQ-TREE v2.0.3.

The phylogenetic relationships between SDSD and SDSE strains were inferred based on the single-copy core genome of these 106 *S. dysgalactiae* genome sequences (Figure 2). This tree was rooted on the largest branch as the closest outgroup species, and *S. pyogenes* was too distant to provide a robust root inference (Supplementary Figure S2). The phylogenetic analysis with *S. pyogenes* was inferred following the same methodology and produced a dataset of 431 single-copy core protein-coding sequences (Supplementary Table S4) shared by the 106 *S. dysgalactiae* and four *S. pyogenes* isolates (Supplementary Figure S2). Both analyses segregated all human SDSE in a single cluster separated from all other animal SD. The animal SDSE strains are more closely related to SDSD strains (Figures 2A,B and Supplementary Figure S2).

**FIGURE 3:**
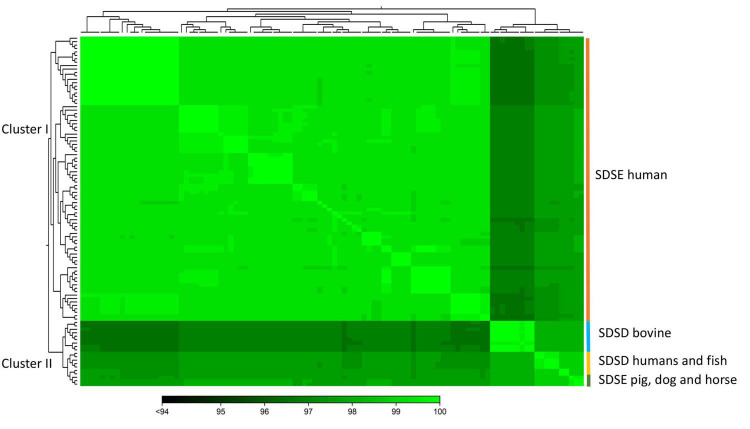
The heatmap chart generated from distances calculated based on the ANI values of *S. dysgalactiae* core genome. The colors in the heatmap represent pairwise ANI values, with a gradient from dark green (low identity) to light green (high identity). The dendrogram directly reflects the degree of identity between genomes increasing in similarity from dark to green. Heatmap and dendrogram of ANI values were performed using the CLC Genomic Workbench.

To quantify the genomic similarities among isolates, we generated a dendrogram and a heatmap with the ANI values of all *S. dysgalactiae* using the core genome dataset (Figure 3). All within-group and among group comparisons are above 96%, indicating the high similarity of these genomes. Similar to the phylogenetic analysis, ANI values segregate *S. dysgalactiae* genomes into two main groups: cluster I, comprising the human SDSE, and cluster II comprising the animal SDSE and the SDSD isolates. Interestingly, the latter can be further subdivided based on the host isolates. All SDSD bovine strains are segregated into a single group within cluster II, while the second subcluster is subdivided into two groups separating humans and fish SDSD isolates and horse, dog, and pig SDSE isolates. The ANI analyses reinforce that bovine SDSD comprises a very homogenous group separated from the remaining strains.

### Accessory genome characterization

**FIGURE 4:**
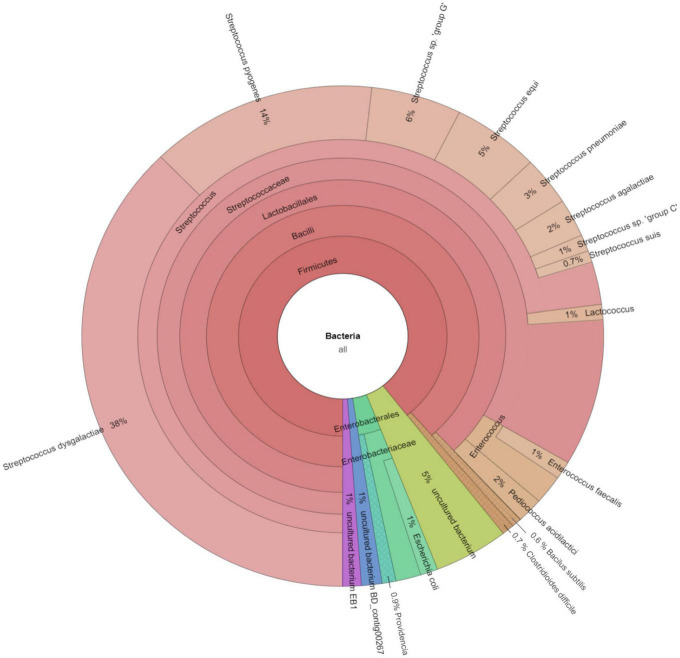
Metagenomics analysis of the accessory genome of *S. dysgalactiae*. CCMetagen graph shows the percentage of accessory genes shared with other bacteria. The alignment of accessory genes against the GenBank database using BLAST was performed to trace homolog genes using the CCMetagen analysis tool. The figure and legend were automatically generated based on the nomenclature deposited in the NCBI.

The software CCMetagen was used to investigate the possible origin of accessory genes by screening with the NCBI database (Figure 4). CCMetagen results suggest that 38% of the accessory genes of *S. dysgalactiae* are only found in this species. These are putative orphan genes (or errors). Around 14% of the accessory genome is shared with *S. pyogenes*, 18% is shared with other species of the genus *Streptococcus*, and approximately 20% of the genes are shared with other bacteria. Interestingly, *S. dysgalactiae* has been found to share genes with phylogenetically distant bacteria, such as the Enterobacteriaceae.

**FIGURE 5:**
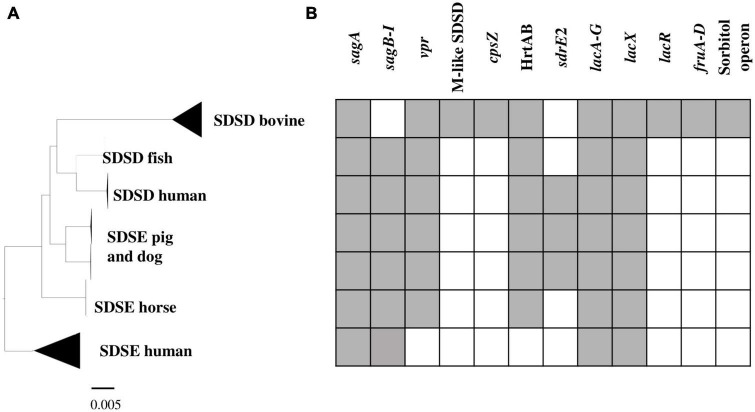
Virulence and carbohydrate metabolism profiles of the 106 *S. dysgalactiae* isolates. **(A)** Phylogenetic analysis of the *S. dysgalactiae* single-copy core genome as in Figure 2. **(B)** Graphic representation of the presence (gray) and absence (white) of the identified relevant gene clusters in the *S. dysgalactiae* genomes in this study. *sagA* to *sagI* – Streptolysin (SLS) operon; *vpr* gene – C5a peptidase*;* M protein-like (SDSD); *cpsZ* gene – Emm-like cell surface protein; *sdrE2* – adhesin; HrtAB – Heme efflux system; *lacA* to *G, lacX and lacR* genes – lactose metabolization operon; *fruA* to *D* – fructose metabolism operon; Sorbitol operon – Sorbitol/glucitol metabolism operon. The complete set of data is shown in Supplementary Table S9.

To investigate genes/operons that may be related to host adaptation, we compared the *S. dysgalactiae* accessory genome from different hosts. Thus, virulence genes, carbohydrate metabolism profiles, and antimicrobial and metal resistance determinants of each *S. dysgalactiae* accessory genome were analyzed (Supplementary Table S5). Our results reveal that, although most accessory genes of *S. dysgalactiae* are widely distributed among isolates from different hosts, some genes seem to be associated with distinct clusters (Figure 5 and Supplementary Table S5). Some of these genes/operons were found to be unique for bovine SDSD isolates, e.g., fructose and sorbitol metabolism operons and genes encoding M-like proteins. Additionally, several genes were shared between all SDSD isolates and SDSE from host animals (non-humans), e.g., genes encoding heme efflux system ATPase (*hrtA* and *hrtB* genes) and *vpr* gene.

Regarding antibiotic and metal resistance profiles, no differences were observed between groups (Supplementary Table S5). Interestingly, genes coding for β-lactamases and VanZ were identified in the *S. dysgalactiae* genome. We compared the sequences coding for β-lactamases enzymes (CDS: WP226316797.1 and WP226316205.1) and glycopeptide resistance functional domain and glycopeptide resistance (*vanZ* gene, CDS: WP003049062.1) sequences from the NCBI database using the BLAST Tool. Our analyses reveal that these genes are highly conserved among strains belonging to the pyogenic group of *Streptococcus*, with an identity greater than 70%.

Nine genes associated with the operon for lactose metabolism (*lacABCDEFGX*) were found in both subspecies; however, the *lacR* gene (lactose phosphotransferase system repressor) was only found among bovine SDSD strains. The *lacR* gene was just downstream of the nitrogen regulatory protein P-II and fructose metabolism operon, found exclusively in bovine SDSD isolates. BLAST analysis revealed that the operons for lactose and fructose metabolism of bovine SDSD shared a high identity (>98%) with the homologous region present in *S. agalactiae* NCTC8184. The operon for sorbitol/glucitol metabolism was also found exclusively in bovine SDSD genomes.

The *cspZ* gene (Emm-like cell surface protein, CDS: VDZ39525.1) was found exclusively in bovine SDSD isolates. This gene shares greater than 70% identity between bovine SDSD and *Streptococcus equi.* Among the bovine SDSD genomes, the *mga* gene and an open reading frame (ORF) previously identified as homologous to *S. pyogenes* M proteins (Porcellato et al., 2021) were observed upstream of the *cspZ* gene. The *mga* gene of bovine SDSD shares approximately 70% identity with *S. pyogenes*, but the ORF is around 40% identical to the *S. pyogenes* sequence. To confirm this homology, we performed a structured-based alignment of the predicted amino acid sequences against the PDB database. Our results corroborate the M protein identification for five SDSD genomes in our dataset (Table 2). Sequences from VSD22, VSD45, and NCTC4670 SDSD isolates produced significant results against chain M of 2XNX PDB structure that corresponds to bc1 fragment of streptococcal M1 protein, while sequences from FDAARGOS1157 and NCTC4671 strains had significant results against chains B and D, respectively of 2OTO structure PDB matching the N-terminal fragment M1 protein (Table 2 and Supplementary Figure S3). The alignments performed with VSD9, VSD16, and NCTC4669 did not retrieve any significant results in the PDB database, but their ORF contains a conserved peptide signal homologous to the one present in M protein of the *S. pyogenes* (Supplementary Figure S4). A second homolog (DemA, CDS: CAB65411.1) of the M and M-like protein was found restricted to bovine SDSD genomes.

**TABLE 2:** Protein Data Bank (PDB) identification of the M-like protein sequence from bovine SDSD isolates.

Isolates	PDB structures	Chain	*E*-value	% Match identity	Resolution (Å)
SDSDVSD22	2XNX	M	2.82E-8	63.64	3.30
SDSDVSD45	2XNX	M	2.91E-8	63.64	3.30
SDSDNCTC4670	2XNX	M	2.93E-8	63.64	3.30
SDSDNCTC4671	2OTO	D	7.26E-7	53.03	3.04
SDSDFDAARGOS1157	2OTO	B	6.87E-7	53.03	3.04

(1) E-value measures the quality of the match from the BLAST search; (2) % Match identity is the identity between the query sequence and the BLAST hit in the matched region; (3) Resolution of crystal structure.

**FIGURE 6:**
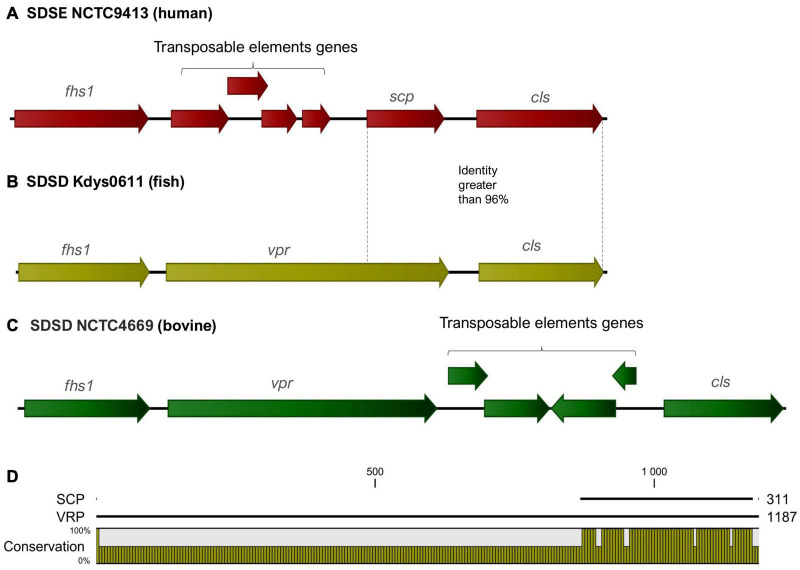
**(A–C)** Graphical representation of the organization of the region flanking the gene that codes for the C5a peptidase. The *fhs1* and *cls* genes encoding formate–tetrahydrofolate ligase and cardiolipin synthetase, respectively, shared an identity greater than 95% among *S. dysgalactiae* strains. The gene identified as *vpr* (C5a peptidase precursor ScpZ) in strain SDSENCTC4669 (CDS: VDZ40442.1) is shared among SDSD animals SDSE strains, while *scp* gene (C5a peptidase) of the SDSENCTC9413 strain (CDS: VTT04007.1) is shared among human SDSE strains. **(D)** BLASTp alignment of predicted proteins VPR and SCP suggests that *scp* is a fragment of the *vpr* gene and reveals that the region coding for the C5a peptidase is absent from the *scp* gene.

In SDSD and animal SDSE isolates (pig, dog, and horse host), the *vpr* gene was found to share an identity greater than 95%. BLAST_*P*_ alignment of predicted protein indicates that *vpr* encodes peptidase S8 family domain in Streptococcal C5a peptidases, protease, and adhesin/invasin. The LP_*X*_TG-motif cell wall anchor domain was also identified. This C5a peptidase is flanked by the *fhs1* and *cls* genes encoding formate–tetrahydrofolate ligase and cardiolipin synthetase, respectively, and shared an identity greater than 95% among *S. dysgalactiae* isolates (Figure 6). Results also show that the *scp* gene found among human SDSE isolates (CDS: VTT04007.1) was found to share > 90% identity (overlap of around 40%) with the *vpr* gene (Figure 6). BLASTp alignment of predicted protein SCP indicates the presence only of the LP_*X*_TG-motif cell wall anchor domain, suggesting that this gene may have been disrupted by mobile genetic elements in the human SDSE lineage (Figure 6).

The adhesin *sdrE*_2 gene (CDS VTT00995.1) shared an identity greater than 85% among animal SDSE and human SDSD isolates. This adhesin belongs to MSCRAMM (acronym for “microbial surface components recognizing adhesive matrix molecules”) family SdrC/SdrD. Features of this protein family include a YSIRK-type signal peptide at the N-terminus and a variable-length C-terminal region of Ser-Asp (SD) repeats followed by an LP_*X*_TG-motif. The *sdrE*_2 gene was found in genomic regions with insertion sequences, commonly associated with signals of horizontal gene transfer, and flanked by homologous regions among S. *dysgalactiae* isolates. The structure of the genomic regions flanking the *sdrE*_2 gene is shown in Supplementary Figure S5.

#### *Streptococcus dysgalactiae* prophages regions

Prophage regions were found on all genomes analyzed. No differences were found in the number of prophage regions among the different groups of isolates, except for the fish SDSD isolates. The fish SDSD Kdys0611 and SDSD STREP9715 isolates were found with the highest number of regions of prophages per genome, with 12 and 23 regions, respectively (Supplementary Table S6). Overall, 72 of the prophage regions were considered intact, 68 were considered questionable, and 310 prophage regions were considered incomplete (Supplementary Table S6). Since several of the SD genomes analyzed are contigs, some regions of prophages may have split into different contigs, and only complete sequences of intact prophages were analyzed.

The intact regions of *S. dysgalactiae* prophages ranged from 19.4 to 87.7 Kb, with 24 and 112 protein-coding regions, respectively. Based on the identity of coding regions within intact prophages, PHASTER indicates that the most prevalent intact prophages corresponded to prophages initially identified in *S. pyogenes* (Supplementary Table S6), such as Strept_315, Strept_phi3396, Strept_phiNJ2, Strept_T12, Strept_P9, and Strept_A25 (Supplementary Table S6).

To identify the intact prophage and screen similar prophage regions, a blast against the NCBI database sequences was also performed (Supplementary Table S7). The result of sequence similarity analysis showed that approximately 69% (50/72) of the intact prophages are unknown (Supplementary Table S7). Around 30% (22/72) of the prophage regions found in the genomes studied in the present study showed overlap greater than 60% and nucleotide identity greater than or equal to 80% with known phage genomes (Table 3 and Supplementary Table S7). Most intact prophages are shared between genomes of the pyogenic streptococcus group. A total of 3 and 7 prophages were found only among SDSD and SDSE, respectively; 8 prophages were shared only by *S. dysgalactiae* genomes; and 42 prophages were shared by the *S. dysgalactiae* and *S. pyogenes* genomes (Supplementary Tables S7, S8). *S. dysgalactiae* prophage regions were also found to share a high identity with *S. agalactiae*, *S. canis, S. pyogenes, S. equi* subsp. *Equi*, *S. parauberis*, and *Lactococcus garvieae* prophage regions (Table 3 and Supplementary Table S8).

**TABLE 3:** Distribution of the Abi and RM systems and non-specific endonuclease of *S. dysgalactiae.*

	Proteins	Frequency* (%)	Function/Description	Strains	RefSeq/BLAST nt	Other species
Abi	AbiEi	26.08	Interfering with transcription	*S. dysgalactiae*	CCW38134.1	*S. agalactiae, S. suis, S. pluranimalium*
	AbiEii	25.21			CCW38135.2	
	AbiSD	9.56	Unknown	Bovine *S. dysgalactiae*	YP_006081091.1	–
RM	YeeAM	8.69	Type I	Bovine *S. dysgalactiae*	WP_003049571.1	*S. equinus, S. pseudopneumoniae, S. mitis, S. suis, S. parasuis*
	YeeAR				WP_011527759.1	
	HsdM	0.87	Type I	SDSDKdys0611 and SDSDSTREP9715	WP_002265322.1	*Lactococcus lactis* subsp. *lactis, Leuconostoc mesenteroides* subsp. *mesenteroides, Lactococcus raffinolactis*
	HsdR				QGH04647.1	
	Subunit M	1.73	Type III	SDSDDB49998-05	WP_016175772.1	*Enterococcus gilvus, Enterococcus faecalis, Lactococcus raffinolactis*
	Subunit R				WP_016175771.1	
Non-specific endonuclease	Spd3	8.69	Streptococcal phage DNase protein	*S. dysgalactiae*	CP_043530.1	*S. pyogenes, S. canis, S. equi*
	Streptodornase B	11.3	Mitogenic factor phage DNase protein	Bovine *S. dysgalactiae*	YP_008243972.1	*S. pyogenes*
	Streptodornase D	100	Streptococcal phage DNase protein	*S. dysgalactiae*	WP_012766664.1	*S. pyogenes, S. equi*

#### Phage resistome analysis

To better comprehend the phage–host interplay among *S. dysgalactiae*, the screening of abortive infection (Abi) and restriction–modification (RM) systems, and non-specific endonuclease among accessory genomes were performed. The results of this analysis suggest that the phage resistome-associated sequences are widely distributed throughout *S. dysgalactiae* strains. Table 4 summarizes the *S. dysgalactiae* phage resistome.

**TABLE 4:** Prophage genome identification using BLASTn at NCBI.

Genome	Phage (ID. PHASTER)	Blast NCBI	Host
SDSD ATCC 27957	Strept_315.3B	S. phage Javan119	SDSD
SDSD DB6070515	Strept_phiNJ2	S. phage Javan88	SDSD, SDSE, *S. canis, S. pyogenes*
		S. phage Javan91	
SDSD NCTC13731	Strept_315.4	S. phage Javan157	SDSD, SDSE, *S. pyogenes*
SDSD STREP9715	Strept_phiNJ2	S. phage Javan91 or	SDSD, SDSE, *S. canis*, *S. pyogenes*
		S. phage Javan88	
SDSE 167A	Strept_phi3396	S. phage Javan133	SDSE, SDSD
SDSE ASDSE99	Strept_315.3	S. phage Javan117	SDSE
SDSE GGS124	Strept_315.3	S. phage Javan144	SDSE, *S. pyogenes*
SDSE KNZ15	Strept_phi3396	S. phage Javan137	SDSD, SDSE, *S. canis, S. pyogenes*
SDSE KNZ16	Strept_phi3396	S. phage Javan143	SDSD, SDSE, *S. canis, S. pyogenes*
SDSE NCTC10238	Strept_T12	S. phage Javan128	SDSD, SDSE
	Lister_2389	S. phage Javan274	SDSD, SDSE
SDSE NCTC11555	Strept_315.5	S. phage Javan510	SDSD, SDSE, *S. pyogenes*
SDSE NCTC11564	Strept_A25	Siphoviridae phage	SDSE
	Strept_315.4	S. phage Javan470	*S. pyogenes*
SDSE NCTC13762	Strept_T12	S. phage Javan471	SDSE, *S. pyogenes*
	Strept_phi3396	S. phage Javan117	SDSD, SDSE
SDSE NCTC9414	Strept_315.6	S. phage Javan135	SDSD, SDSE, *S. canis, S. pyogenes, S. equi* subsp. *Equi*
SDSE S6020	Strept_315.3	S. phage Javan150	SDSE, *S. pyogenes*
SDSE UT4234	Strept_315.4	S. phage Javan88	SDSD, SDSE, *S. canis, S. pyogenes, S. equi* subsp. *Equi*
SDSE UT5345	Strept_315.5	S. phage Javan166	SDSE, *S. pyogenes*

We identified the AbiE and AbiSD systems among the *S. dysgalactiae* strains. The AbiE system is organized in a bicistronic operon, encoding the AbiEi toxin and AbiEii antitoxin, while AbiSD has not been fully understood yet. The alignment of *abiEi, abiEii*, and *abiSD* sequences of *S. dysgalactiae* against the GenBank database was performed using BLAST. The analysis revealed that *abiEi and abiEii* are present in the genome of *S. agalactiae*, *S. suis*, and *S. pluranimalium*, and *abiSD* is present in the genome of bovines’ SDSD strains.

AbiEi and AbiEii proteins of the *S. dysgalactiae* strains share high amino acid identity (>87%) with *S. agalactiae* sequences. The high sequence identity of AbiEi (96%) was observed between VSD22 and SDSD NCTC4670 strain. Interestingly, the bovine SDSD NCTC4670 strain does not harbor the *abiEii* gene (Supplementary Figure S6).

Several non-specific extracellular nucleases were identified in the accessory genome of *S. dysgalactiae*. While the Spd3 and streptodornase D nucleases seem to have ubiquitous distribution within the *S. dysgalactiae*, it is also present in other species of *Streptococcus*, such as *S. canis, S. equi*, and *S. pyogenes.* The streptodornase B nuclease (*spd1*) was found only in bovine SDSD isolates and was associated with the putative prophage found in *S. pyogenes* genomes.

The *spd1* gene has been found associated with the *speC* gene (streptococcal pyrogenic exotoxin). Studies carried out by our research team showed that *speC* and *spd1* genes are present in bovine SDSD from different geographic regions and years of isolation, namely SDSD from bovine mastitis isolated in Portugal in 2002–2003 and 2011–2013 (Rato et al., 2013; Alves-Barroco et al., 2021).

In the present work, we have identified *speC-spd1* genes only in putative prophages of the bovine SDSD, in particular, SDSDNCTC4669 (isolated in 1935, London) and SDSDVSD16 (isolated in 2002, Portugal) with high nucleotide identity (greater than 97%). In bovine SDSD prophages, another streptococcal pyrogenic exotoxin gene (*speK*) downstream of the *speC-spd1* region was identified. While the *speC-spd1* genes were originally identified in the M1 phage (from *S. pyogenes*), *speK* was identified in the M3 phage (from *S. pyogenes*), suggesting poly-lysogeny and stable genetic linkage of the *speC*-*spd1*-*speK* genes among bovines SDSD isolates.

Regarding the restriction–modification (RM) systems of the accessory genome of *S. dysgalactiae*, the putative DNA methyltransferase YeeAM and the respective restriction enzyme (herein referred to as YeeAR) were identified only among bovine SDSD strains. However, an analysis of the NCBI database reveals that these enzymes are present in *S. equinus, S. pseudopneumoniae, S. mitis, S. suis*, and *S. parasuis.* The Hsd RM system was found only in fish SDSD strains and was shared with closely related genera such as *Lactococcus* and *Leuconostoc*, whereas the type III RM system was found only in the human SDSD. SDSDDB49998-05 strain was also detected in *Enterococcus* and *Lactococcus.* Interestingly, DNA modification methylase (CDS QBX23397.1 of S. phage Javan128) was found in all putative Strep_12 prophages identified in this study.

## Discussion

This study provided an analysis of genomic features of SD associated with specific hosts. The phylogenetic relationship of SD strains showed that human and animal isolates are segregated into two clearly distinct groups. Supporting our results, ANI pairwise comparison of the core genome grouping SD isolates into two main clusters: cluster I harbors only human SDSE isolates, while cluster II harbors SDSD and animal SDSE isolates (Figure 3), corroborating the groups detected by phylogenetic analysis.

Our phylogenetic relationship analysis and the ANI values of the comparison of isolates revealed that human and fish SDSD are closely related. These results corroborate the phylogenetic relationships based on the *sodA* gene sequences (Koh et al., 2020). Koh et al. (2020) findings suggest that the source of infection in humans by SDSD was suspected to be the fish.

Additionally, Koh et al. (2020) reveal that the human SDSD isolates were isolated from blood cultures of human patients with a similar clinical presentation: DB6070515 (isolated in 2015) from a patient with breast cancer; DB5399317 (isolated in 2017) from a patient with breast cancer, and DB4999805 (isolated in 2005) from a patient who had a partial mastectomy. This patient developed septicemia after a cut on her hand while cleaning fish (Koh et al., 2020). Apparently, the compromised immune system of those patients favored infections associated with SDSD isolates. However, more detailed research into the isolation of human SD is needed to assess whether it is a carrier phenomenon or whether the species can be permanently integrated into the human microbiome, making it ready to cause opportunistic infections.

Currently, the taxonomic status of subspecies SD remains controversial despite years of debate and reclassifications. In routine human and veterinary clinical laboratories SDSD and SDSE have been distinguished based on their hemolytic properties on blood agar (Jensen and Kilian, 2012). Among the pyogenic group of streptococci, the hemolytic activity is mainly attributed to streptolysin S (SLS) production. The operon encoding SLS includes the prepropeptide structural gene (*sagA*), followed by genes responsible for converting SagA into SLS, for transport, and the leader cleavage (*sagBCDEFGHI*). Datta et al. (2005) reported that all genes of the *sag* operon are required for the expression of functional Streptolysin S. Furthermore, previous studies revealed that mutations in the core region of *sagA* cause the loss of β-hemolytic activity (Molloy et al., 2015).

We previously reported that differences in hemolytic patterns between human SDSD and SDSE (β-hemolytic) and bovine SDSD isolates (α-hemolytic) may be related to the loss of *sagB-I* genes observed in bovine SDSD isolates (Alves-Barroco et al., 2021). According to the present study’s analysis, the Streptolysin S operon is present in the animal cluster but absent in the bovine isolates.

Together, the data suggest that hemolytic profiles are not a good indicator for the taxonomic classification of subspecies SD. Thus, the failure to distinguish SDSD from SDSE in routine laboratory tests underestimates the exact incidence of SDSD human infections.

To determine the robustness of the phylogenetic relationships observed with the core genome, we analyzed the genome accessory to identify differences among isolates of the different hosts. Interestingly, our results on the presence/absence of some genes of the accessory genome also provide further support for phylogenetic relationships based on the core genome. We found virulence genes, e.g., *hrtA* and *hrtB* encoding heme efflux system ATPase, the *vpr* gene encoding C5a peptidase that is shared by SDSD, and animal SDSE isolates, but they were absent in all human SDSE isolates.

Among human SDSE, the *scpG* gene, encoding C5a peptidase, was found with a high degree of identity with the protein from *S. agalactiae* and *S. pyogenes* (Brandt and Spellerberg, 2009). In the present work, we find that the *vrp* gene contained a functional domain encoding a C5a peptidase not previously described in *S. dysgalactiae*. The *scp* gene that showed an overlap of around 40% with the homologous *vpr* gene is shared by only human SDSE isolates. Transposable elements were found upstream of *scp* gene, suggesting that this gene may have been disrupted by mobile genetic elements in the human SDSE lineage.

*Streptococcus dysgalactiae* harbors a great repertoire of adhesins that interact with the extracellular matrix of animal cells, including SfbI, GfbA, FbaA, FbaB, FBP54, and M-like proteins (Alves-Barroco et al., 2020). Here, we found several adhesins ubiquitously and randomly distributed among *S. dysgalactiae* isolates, except the *sdrE*_2 gene that was found only in animal SDSE and human SDSD isolates.

Since carbohydrate metabolism may be associated with adaptation to different niches, we search for the presence of the lactose, fructose, and sorbitol/glucitol metabolism operons in the accessory genome of *S. dysgalactiae*. While the operons for fructose and sorbitol/glucitol metabolism were restricted to bovine SDSD, the operon for lactose metabolism was widely distributed among *S. dysgalactiae*; however, the *lacR* gene (lactose system repressor) was restricted to bovine SDSD isolates. These data suggest that the operons for fructose and sorbitol/glucitol metabolism may be specific biomarkers for the bovine host. Like *S. agalactiae*, *S. dysgalactiae* also harbors two Lac operons. In *S. agalactiae*, Lac.1 was suggested to be a virulence system and specific for human isolates, while Lac.2 regulates lactose metabolism in bovine isolates (Richards et al., 2011). These results also revealed that the operon for fructose metabolism is present in bovine isolate samples but absent among human isolates. This study suggests a unique fructose metabolism for the bovine *S. agalactiae* isolates, facilitating survival in organs or extramammary tissues (e.g., bovine rumen). Richard and co-workers’ findings suggest the transfer of the operons for fructose and lactose between species causing bovine mastitis. This exchange of genetic material may have provided adaptation to the bovine environment (Richards et al., 2011).

Overall, no differences in antibiotic resistance profiles were observed between the groups. *S. dysgalactiae* has been recognized as non-β-lactamase-producing (Bonofiglio et al., 2018); however, the presence of coding regions for β-lactamase enzymes among *S. uberis* and SDSD genomes recovered from dairy cows in Canada were identified (Vélez et al., 2017). Here, we identified the presence of a genetic determinant for widely distributed β-lactamases among *S. dysgalactiae* of human and animal origins (Supplementary Table S5). However, further studies are needed to assess the potential of these β-lactamases, target antibiotics, and the potential of SD as a reservoir of resistance genes. Although some streptococci strains have been identified as resistant to glycopeptides (Park et al., 2014; Srinivasan et al., 2014; Lai et al., 2017), the mechanisms of resistance to glycopeptides in *Streptococcus* are unclear. The teicoplanin resistance gene (vanZ) was widely distributed among the *S. dysgalactiae* genomes in the present study. The protein encoded by *vanZ* of the *S. dysgalactiae* shares around 57% similarity with *E. faecium* VanZ, indicating the likely origin of the gene.

Our analysis reassesses previous taxonomic identifications and recovers an evolutionary history that suggests that is not compatible with the current taxonomy based on the proposal by Vieira et al. (1998). The phylogenetic relationship showed that human and animal isolates are segregated into two clearly distinct groups. ANI values and characteristics of the accessory genome reinforce the classification based on the phylogeny of the core genome. Thus, we suggest that all isolates in cluster I of Figure 2 should be classified as SDSD, regardless of the hemolytic pattern. However, a more conclusive analysis requires a more sample size of SD isolates sampled from different animal hosts.

Different prophage regions were observed in the *S. dysgalactiae* genomes. Despite the different origins of isolates, most prophages are related. The *S. dysgalactiae* prophage regions display considerable similarity with *S. pyogenes* sequences (around 61%, 51/83), suggesting phage HGT involving these species.

Studies reported the presence of *S. pyogenes* phage-carried virulence genes among bovine SDSD isolates, genes that were not found among SDSE isolates (Rato et al., 2011; Alves-Barroco et al., 2021). Though SDSD and SDSE share homologous regions of prophages, the presence of non-essential genes differentially distributed and different regions of integration of the prophages indicate that HGT events between *S. pyogenes* and both subspecies occurred independently.

Although we did not formally analyze the presence of current gene flow between *S. pyogenes* and SDSD, the results indicated the possibility of gene flow between both. Assuming that SDSD isolates are capable of causing zoonotic disease, it seems likely that they occasionally have shared the same niche favoring the transfer of prophages. A second hypothesis is that the exchange of bacteriophages may have occurred before the ecological niche divergence between the two species, as previously suggested (Alves-Barroco et al., 2021). However, the direction of evolution and gene flow between SDSD and *S. pyogenes* remains to be clarified.

Overall, the data suggest that although bacteriophage resistome-associated systems are widely distributed throughout *S. dysgalactiae*, greater diversity of systems is found in SD isolated from animal sources.

Restriction-modification (RM) system was found to share a high identity with species belonging to another bacterial genus, such as *Lactococcus lactis subsp. lactis*, *Lactococcus raffinolactis*, *Enterococcus gilvus*, and *Enterococcus*. RM systems are considered the most ubiquitous bacteriophage resistance mechanism, and therefore the easiest for phages (Labrie et al., 2010), including the phage-encoded methylases. Here, DNA modification methylase was found in all putative Strep_12 prophages. Besides that, when RM systems fail, the bacteriophages will be replicated and modified by the bacteria cell, becoming resistant to restriction.

In the abortive infection (Abi) system, the resistance results in the death of the phage and the bacteria; therefore, they are “altruistic” cell death systems, protecting the bacterial population. The Abi mechanisms arrest bacteriophage development in different stages, e.g., phage transcription or genome replication (Labrie et al., 2010). Here, we identify the AbiE and AbiSD systems. AbiE is organized in a bicistronic operon, encoding the antitoxin (AbiEi) and toxin (AbiEii). While AbiE is widely distributed throughout *S. dysgalactiae*, AbiSD is restricted to SDSD bovines. Most toxin–antitoxin (TA) systems encode two components, a toxic protein that generally targets essential cellular processes and an antitoxin (Labrie et al., 2010).

Alignment of the *abiEi* and *abiEii* sequences of *S. dysgalactiae* against the GenBank database revealed > 87% identity with the *S. agalactiae* homologous. In *S. agalactiae*, AbiE system functions as a Type IV TA system. AbiEii induces bacteriostasis, while AbiEi can neutralize the AbiEii expression. Furthermore, it was observed that AbiEi negatively autoregulates abiE operon expression; thus, AbiEi is an antitoxin and a transcriptional repressor (Dy et al., 2014). Interestingly, the bovine SDSD NCTC4670 strain does not harbor the *abiEii* gene but has the *abiEi* gene, which likely regulates the expression of other genes besides the AbiE operon.

Here, we did not observe a correlation between the number of prophages regions and resistome systems in the genomes of *S. dysgalactiae*, suggesting that no mechanism of resistance to bacteriophages is completely efficient. Furthermore, bacteriophage genomes are flexible with fast evolution in response to bacterial resistance systems.

## Conclusion

The phylogenetic relationship based on the core genome suggests that *S. dysgalactiae* strains do not cluster by subspecies as it was suggested by Vieira et al. (1998). The phylogenetic analysis and ANI pairwise comparison showed that human and animal isolates are segregated into two distinct groups, supporting the classification proposed by Vandamme et al. (1996). According to the proposal of Vandamme and co-workers, all SD isolates from the animal source must be classified as SDSD. Thus, the existence of an SDSD group that causes human–animal cluster infections may be due to opportunistic infections, and the exact incidence of SDSD human infections may be underestimated due to failures in identification based on the hemolytic patterns. However, more detailed research into the isolation of human SD is needed to assess whether it is a carrier phenomenon or whether the species can be permanently integrated into the human microbiome, making it ready to cause opportunistic infections. The number and diversity of prophage regions in the *S. dysgalactiae* genome enforce the premise that horizontal transfer influences the genomic features, being able to contribute to genetic repertoire and adaptation to changing environments. Features of the accessory genome provide further support for the phylogenetic relationships observed, suggesting a signature of host adaptation and host-specific SDSD population in bovines.

## Data Availability Statement

The datasets presented in this study can be found in online repositories. The names of the repository/repositories and accession number(s) can be found in the article/Supplementary Material.

## Author contributions

CA-B, PB, IS-S, and AF contributed to the idea or design of the research. CA-B performed the genomic DNA extraction, assembly, annotation of the VSD genomes, phylogenetic analysis and pangenome diversity of the *S. dysgalactiae*, and manuscript writing. CA-B, PB, and AF performed the revision and editing of the final manuscript. All authors contributed to the article and approved the submitted version.

## Conflict of Interest

The authors declare that the research was conducted in the absence of any commercial or financial relationships that could be construed as a potential conflict of interest.

## Publisher’s Note

All claims expressed in this article are solely those of the authors and do not necessarily represent those of their affiliated organizations, or those of the publisher, the editors and the reviewers. Any product that may be evaluated in this article, or claim that may be made by its manufacturer, is not guaranteed or endorsed by the publisher.

## Footnotes

1
https://digitalinsights.qiagen.com/products-overview/discovery-insights-portfolio/analysis-and-visualization/qiagen-clc-genomics-workbench/
