# Supplementary material for: Phylogenetic analysis and accessory genome diversity reveal insight into the evolutionary history of Streptococcus dysgalactiae
Source: Front Microbiol. 2022 Jul 19;13:952110. doi: 10.3389/fmicb.2022.952110 (PMC9343751; doi:10.3389/fmicb.2022.952110)
Supplement: Supplementary file 1 [file Data_Sheet_1.zip › Supplementary Figures.docx]

**Phylogenetic analysis and accessory genome diversity reveal insight into the evolutionary history of *Streptococcus dysgalactiae***

Cinthia Alves-Barroco^1,2,^*, Patrícia H. Brito^1,2,3,^*, Ilda Santos-Sanches^1,¥^, Alexandra R. Fernandes^1,2,^*

1. Applied Molecular Biosciences Unit (UCIBIO), Dept. Ciências da Vida, NOVA School of Science and Technology, Costa da Caparica, Portugal
2. i4HB, Associate Laboratory - Institute for Health and Bioeconomy, Faculdade de Ciências e Tecnologia, Universidade NOVA de Lisboa, 2819-516 Caparica, Portugal
3. NOVA Medical School, Faculdade de Ciências Médicas, Universidade NOVA de Lisboa, 1169-056 Lisboa, Portugal

*Corresponding author: [c.barroco@campus.fct.unl.pt](mailto:c.barroco@campus.fct.unl.pt) (CA-B); [phbrito@fct.unl.pt](mailto:phbrito@fct.unl.pt) (PHB), [ma.fernandes@fct.unl.pt](mailto:ma.fernandes@fct.unl.pt) (ARF)

^¥^ In memory of Professor Ilda Santos-Sanches


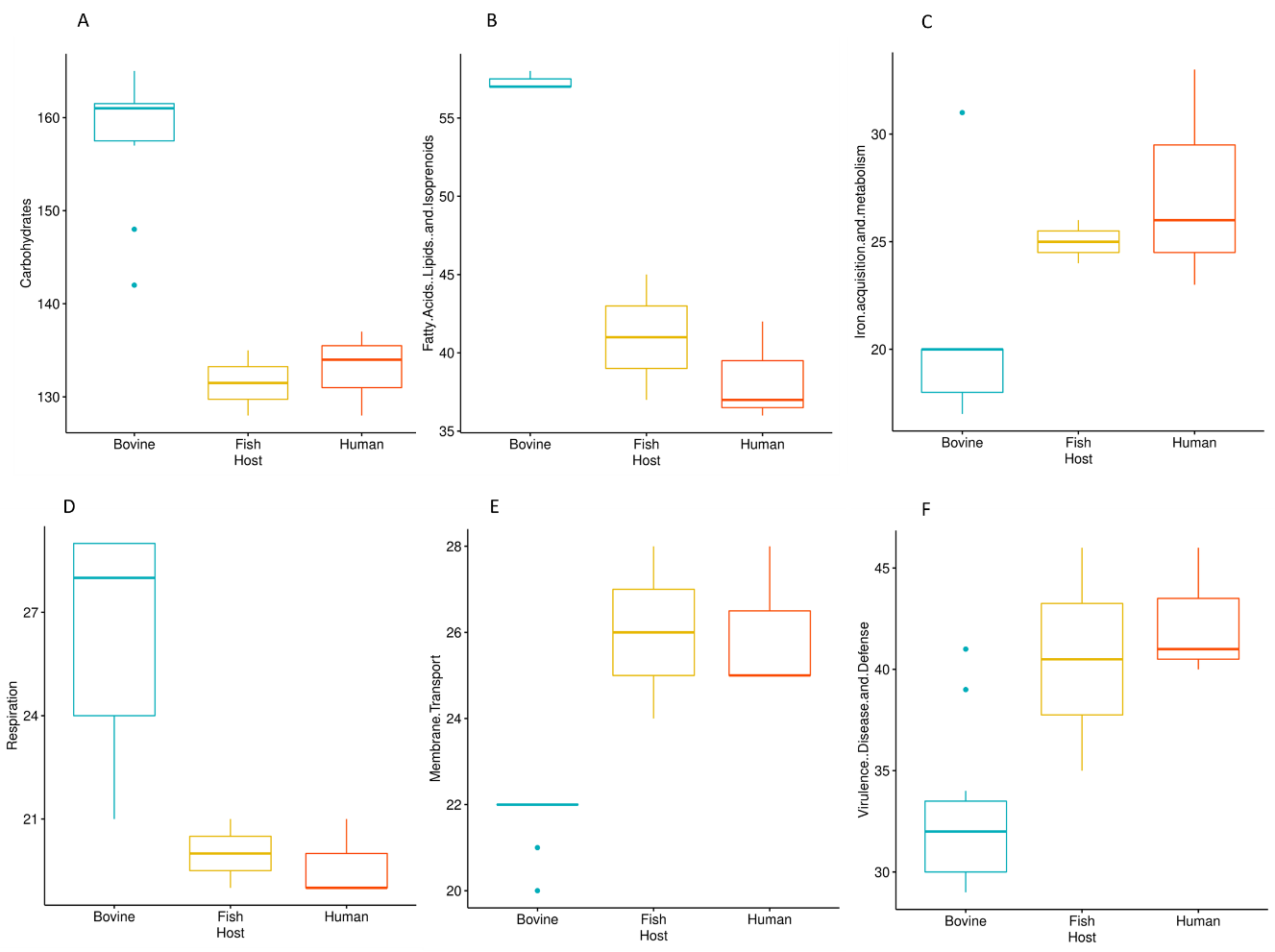


**Figure S1.** Box-plot of the Kruskal-Wallis test for (A) Carbohydrates; (B) Fatty Acids, Lipids and Isoprenoids; (C) Iron acquisition and metabolism; (D) Membrane Transport; (E) Respiration and (F) Virulence and Defense sistems.


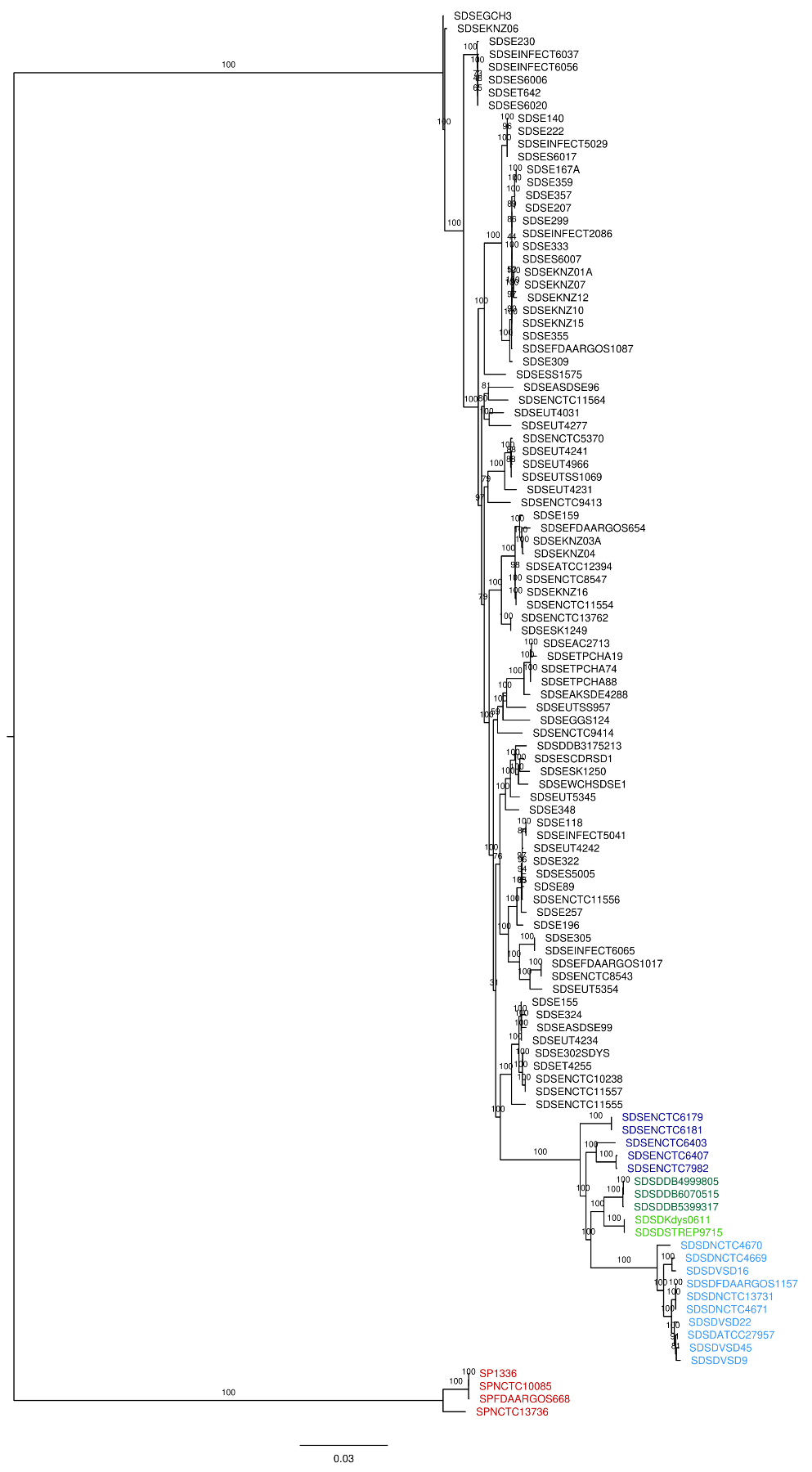


**Figure S2.** The evolutionary history inferred using the Maximum Likelihood analysis of the alignment of 431 single-copy core protein-coding sequences shared by 106 *S. dysgalactiae* and 4 *S. pyogenes* genomes. Bootstrap support values were calculated from 1000 replicates. Black – human SDSE; Dark blue – non-human SDSE, Light blue – SDSD bovine; Light Green – Fish SDSD, Dark Green – Human SDSD; Red – *S. pyogenes.*


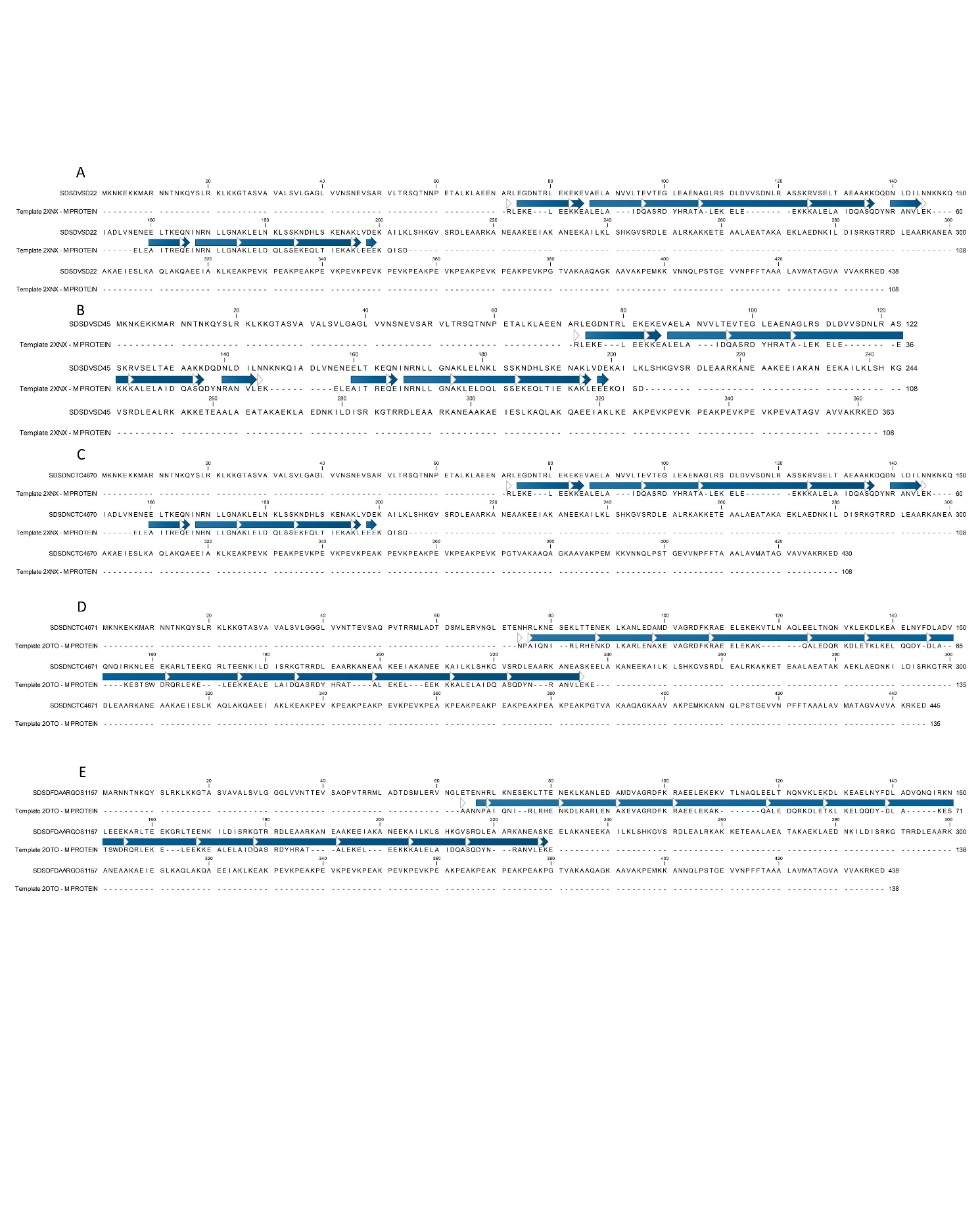


**Figure S3.** Sequences from VSD22, VSD45, and NCTC4670 SDSD isolates produced significant results against chain M of 2XNX PDB structure that corresponds to bc1 fragment of streptococcal M1 protein, while sequences from FDAARGOS1157 and NCTC4671 strains had significant results against chains B and D, respectively of 2OTO structure PDB matching the N-terminal fragment M1 protein.


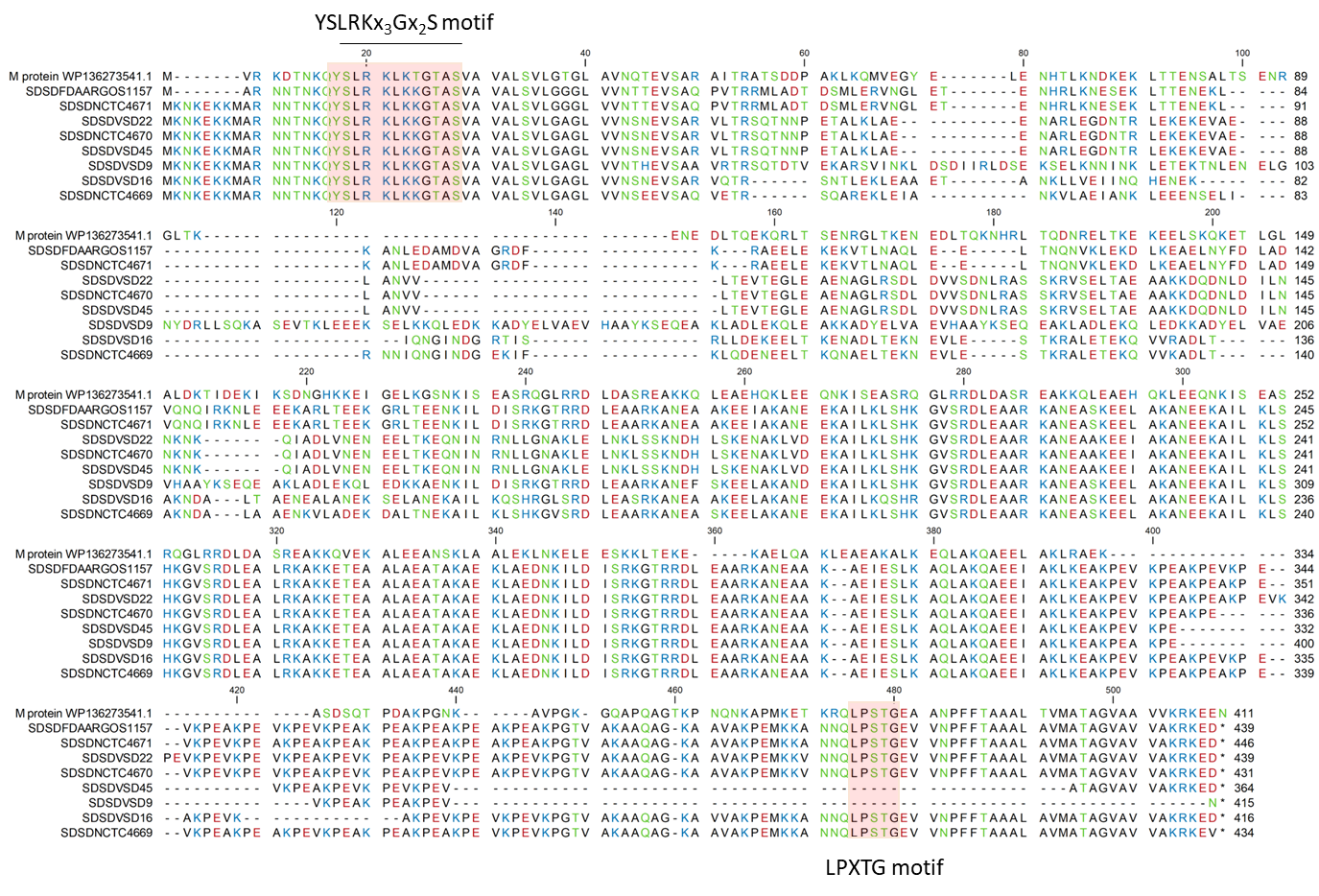


**Figure S4.** Sequence alignment of the M protein of the *S. pyogenes* (NCBI Reference Sequence: WP136273541.1) and bovines SDSD sequences. These sequences include an signal sequence in the N-terminal region that directs the protein for secretion. The YSLRKx_3_Gx_2_S motif contained in the signal sequence conserved among SD and *S. pyogenes* isolates is shown. In the C-terminal region LP_X_TG, the motif was lost in VSD9 and VSD45 bovine isolates.


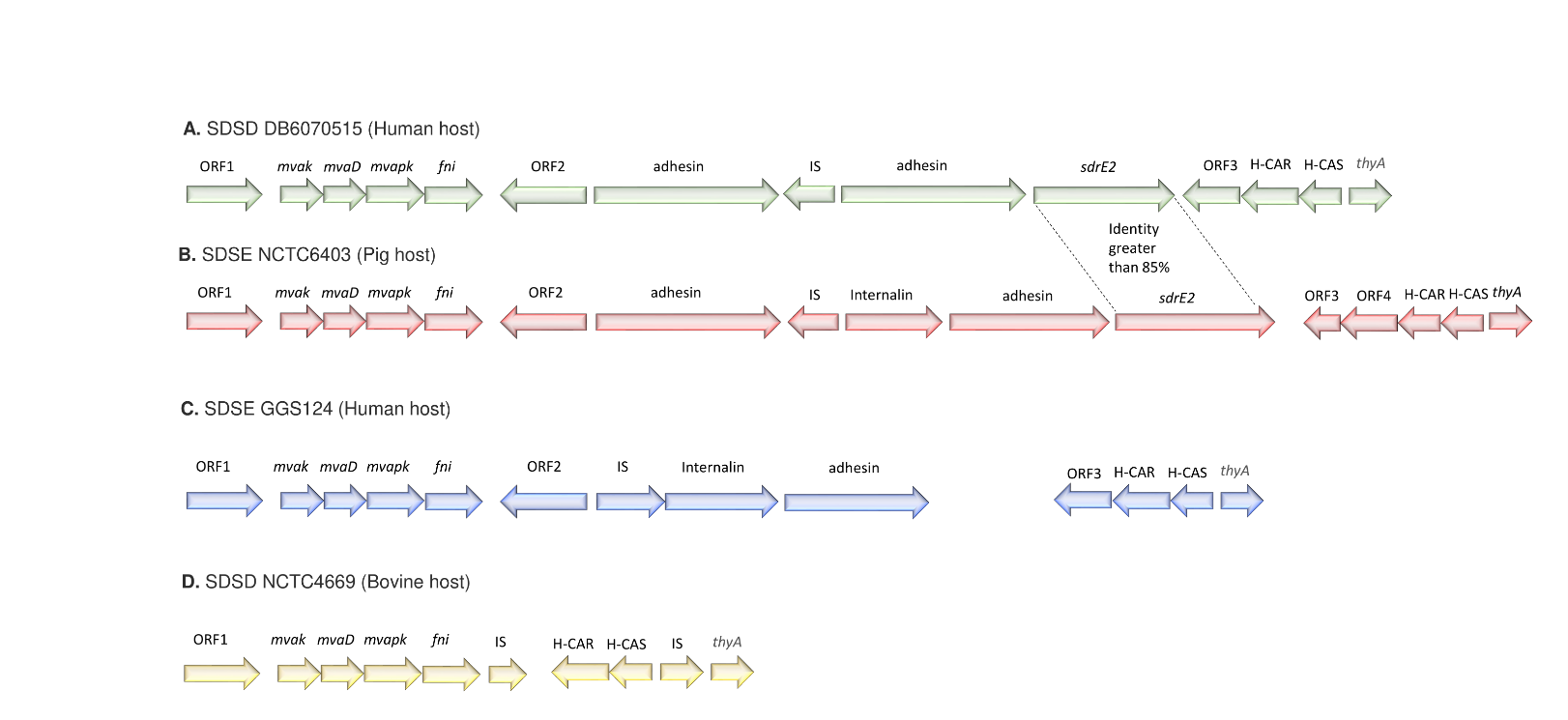


**Figure S5.** Structure of the genomic regions flanking the *sdrE*_2 gene in (A) SDSD DB4999805 and (B) SDSE NCTC6403 isolates. The *sdrE*_2 gene shares an identity greater than 85% among animal SDSE and human SDSD isolates but absent in the other SD isolates. The homologous genomic region of (C) SDSE GGS124 and (D) SDSD NCTC4669 isolates is also shown. The SdrE2 belongs to MSCRAMM family SdrC/SdrD. ORF 1 - ABC transporter ATP-binding; ORF2, ORF3 and ORF4 - putative transcriptional regulator; Adhesin - LPXTG cell wall anchor domain-containing protein; *mvaK* - mevalonate kinase; *mvaD* - diphosphomevalonate decarboxylase; *mvapK* - phosphomevalonate kinase; *fni* - isopentenyl pyrophosphate isomerase; IS – transposase; Internalin; H-CAR - HMG-CoA reductase; H-CAS - HMG-CoA synthase; *thyA* - thymidylate synthase.


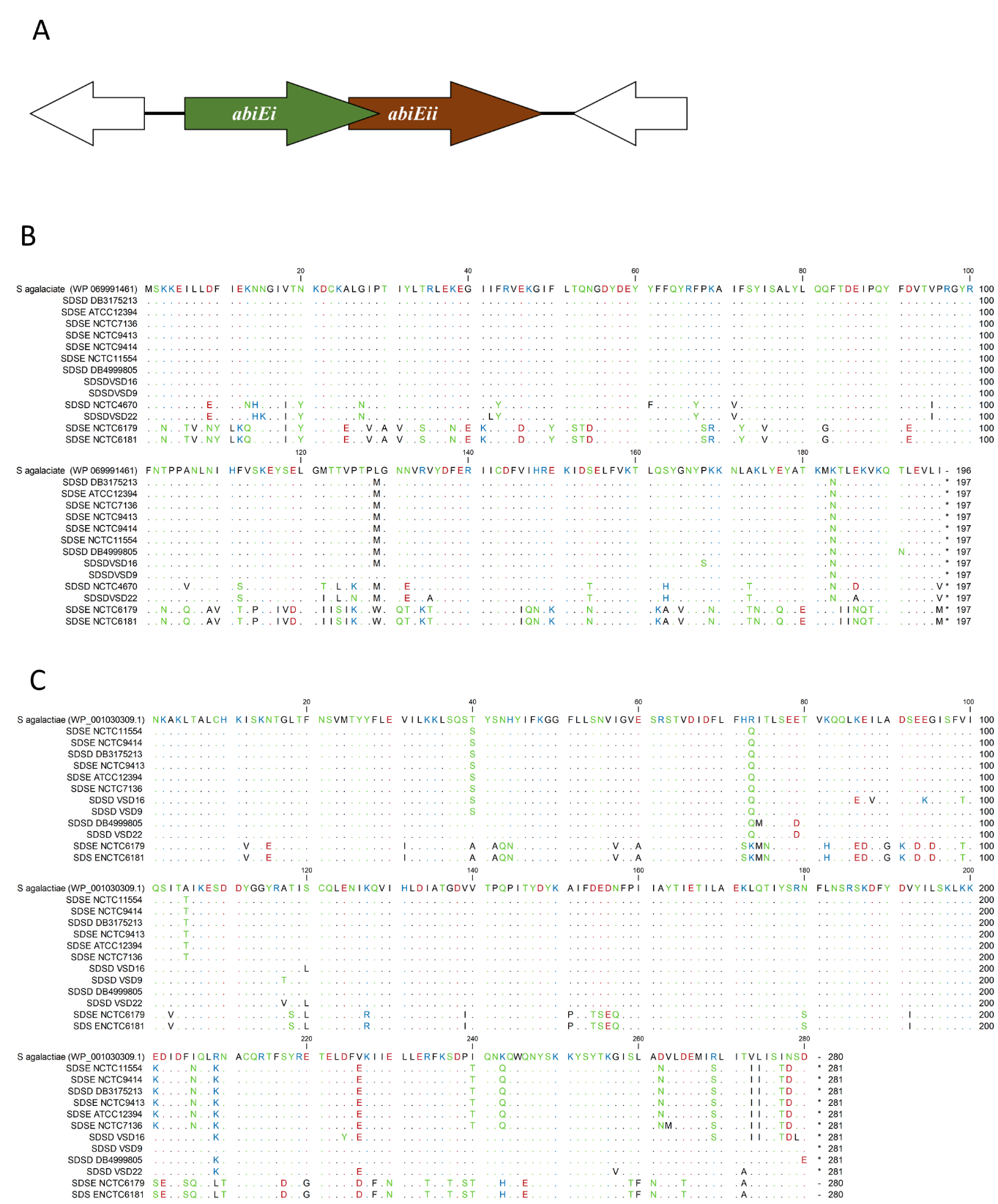


**Figure S6.** (A) Schematic diagram of the genetic organization of *abiE* in the *S. agalactiae* V/R 2603 genome (not to scale). Alignment of AbiEi **(B)** and AbiEii **(C)** peptides. AbiE is organized in bicistronic operon, encoding the AbiEi antitoxin and AbiEii antagonistic toxin. Both proteins share high amino acid identity (>87%) with *S. agalactiae* sequences. The high homology of AbiEi (96%) was observed between VSD22 and SDSD NCTC4670 strain. The NCTC4670 strain seems to have lost AbiEii at some point during evolutionary process. Deduced amino acid sequences from this bovine allele were compared with similar sequences from the NCBI database and were analyzed with the CLC-Genomic Workbench sequence alignment tool.
